# Incidence, dynamics and recurrences of reverse cleavage in aneuploid, mosaic and euploid blastocysts, and its relationship with embryo quality

**DOI:** 10.1186/s13048-022-01026-9

**Published:** 2022-08-05

**Authors:** Lei Jin, Xiyuan Dong, Wei Tan, Bo Huang

**Affiliations:** Reproductive Medicine Center, Tongji Hospital, Tongji Medicine College, Huazhong University of Science and Technology, Wuhan, People’s Republic of China 430030

**Keywords:** Time-lapse, Ploidy status, PGT, Reverse cleavage

## Abstract

**Background:**

During embryonic development, the normality of cleavage and the ploidy state are closely related to the final clinical outcome. At present, many research teams are focusing on the combined application of timelapse (TL) technology and preimplantation genetic testing (PGT) technology, hoping to find a connection between the two aspects of morphodynamics and genes. In the process of embryonic cleavage, there is a common abnormal cleavage pattern called reverse cleavage (RC). RC refers to blastomere fusion and failed cytokinesis. There are very few reports about it. Whether the occurrence of RC affects blastocyst euploidy is even less clear. Whether the RC phenomenon affects the embryonic developmental potential and whether it is related to the embryo ploidy. This is important for clinicians and embryologists. In this study, we used TL to observe whether there was a phenomenon of RC in each biopsy embryo and then combined it with the ploidy state to give an answer, which provided support for the selection strategy of RC embryos.

**Methods:**

A total of 405 TL-PGT cycles and 1,467 blastocysts were included in the study. All TL data were collected from the Reproductive Medicine Center, Huazhong University of Science and Technology Hospital. Embryos images throughout embryonic development, from post-insemination to day 5 or 6 until biopsy and cryopreservation, were acquired by the Embryoscope Plus TL microscopy system from January 2019 to December 2020. This study investigated the overall incidence of RC during cleavage; the relationship between RC phenomenon and the number of occurrences and ploidy results; the relationship between RC occurrence and blastocyst developmental quality, as well as the dynamics of RC embryos.

**Results:**

Among the 1,453 blastocysts biopsied, 400 blastocysts showed RC phenomenon at the cleavage stage, and the incidence rate was 25.9%. In euploid, mosaic and aneuploid embryos, the incidence of RC was 27.2%, 26.6%, and 25.0%, respectively. The incidence of RC was similar among these three groups with no significant difference (*P* > 0.05). The number of RC occurrences was not associated with embryo ploidy status (*P* > 0.05). In general, the blastocyst quality of the RC + group was lower than that of the RC- group. In the ICM score, the proportion of A score in the RC + group was significantly lower than that in RC- group (*P* < 0.05). In the TE score, there was no significant difference between the two groups of A-grade blastocysts, but the proportion of B-grade blastocysts in the RC + group was significantly lower than that in the RC- group (*P* < 0.01). In terms of developmental kinetic parameters, the cleavage synchrony parameters s2 and s3 were significantly longer in RC + embryos than in RC- embryos (*P* < 0.05). However, these changes in kinetic parameters were not significantly different between the euploid, mosaic and aneuploid groups.

**Conclusions:**

The chromosomal euploidy of cleavage-stage embryos with RC phenomenon developed to the blastocyst stage was not significantly different from that of cleavage normal blastocysts. Therefore, RC embryos should not be discarded. It is recommended to select and utilize blastocyst culture, which has similar clinical value to normal cleavage embryos.

**Supplementary Information:**

The online version contains supplementary material available at 10.1186/s13048-022-01026-9.

## Introduction

The selection of potential embryos has always been one of the important topics in the field of assisted reproductive technology (ART) [1]. Time-lapse (TL) embryo culture monitoring system and preimplantation genetic test (PGT) are important methods to help embryo selection in clinical applications [2–4]. A TL embryo culture monitoring system is different from traditional embryo observation methods, which can observe the morphology of embryos at various developmental stages in real time [5–7]. It can obtain the time parameters of various cleavage moments during the embryo cleavage process and can also observe the information on normal cleavage and abnormal cleavage patterns, which may be closely related to the developmental potential of the embryo [8]. On the other hand, PGT technology can know the developmental potential of embryos from the ploidy state of embryos. The developmental potential of aneuploid embryos is far inferior to that of euploid embryos [9–12]. Many research groups are now studying the relationship between embryo information obtained by TL technology and embryo ploidy results [13–15]. Among these research contents, the abnormal cleavage pattern of embryos is an important one. Studies have found that abnormal cleavage directly affects the genome of a single cell in human embryos, resulting in chromosome loss and mosaicism, thereby reducing embryonic developmental potential, resulting in embryonic developmental arrest and significantly reduced blastocyst formation rates [16–18].

Reverse cleavage (RC), which is an abnormal cleavage pattern we encounter from time to time in the clinical embryo selection process. It has been reported that RC can reduce embryonic developmental potential [19–21], and other scholars hold opposing views [22–25]. Nonetheless, for clinicians and embryologists, we still know very little about reverse cleavage, and many embryologists wonder if it will be related to embryonic developmental potential, thereby affecting the ploidy state of the embryo, and further affecting the clinical outcome? Answering these questions requires a combination of embryonic TL information and PGT results.

In this study, we used TL to observe whether there was a phenomenon of RC in each biopsy embryo and then combined it with the ploidy state to give an answer, which provided support for the clinical selection strategy of RC embryos.

## Materials and methods

### Study design and participants

In this cohort study, a total of 405 TL-PGT cycles and 1467 blastocysts were included in the study. All TL data were collected from the Reproductive Medicine Center, Huazhong University of Science and Technology Hospital. Embryos images throughout embryonic development, from post-insemination to day 5 or 6 until biopsy and cryopreservation, were acquired by the Embryoscope Plus time-lapse microscopy system (Vitrolife, Denmark) from January 2019 to December 2020.

All patients signed a written notice of consent and received routine clinical care at this center. No additional intervention were performed. The study complies with the Declaration of Helsinki for Medical Research Involving Human Subjects. This study was approved by the Ethics Committee of the Reproductive Medicine Center of Tongji Hospital.

### Insemination and embryo culture

All PGT cycles included in this study were fertilized by intracytoplasmic sperm injection (ICSI). The specific procedure of ICSI has been described previously [26]. Briefly, before ICSI, the corona radiata and cumulus cells of oocytes were removed by exposure to hyaluronidase; oocyte maturation was then observed under an inverted microscope. ICSI was performed on mature oocytes (metaphase II oocytes). Immediately after injection, all injected oocytes were placed in a time-lapse incubator (Embryoscope Plus). The fertilized oocytes were then cultured continuously for more than 2 days at 6% CO_2_, 5% O_2_, and 37ºC. Check all embryos on the morning of day 3 after oocyte retrieval. Embryos were then changed to blastocyst medium and culture continued until day 5/6. The inner cell mass (ICM) and trophectoderm (TE) of blastocysts were graded according to Gardner [27]. On day 5/6, biopsies of the formed usable blastocysts were performed. Using a laser to make a small hole in the zona pellucida before biopsy, 3–6 trophectoderm cells are obtained by mechanical dissection.

### Time-lapse monitoring and morphokinetic parameters

A total of 4,551 mature oocytes underwent ICSI and were cultured at 37 °C until day 5 or 6 under 6% CO2, 5% O2, and 89% N2 conditions in the TL system, with each embryo at eleven layers images were taken every 10 min at a distance of 150 μm in-depth, roughly the same diameter as an oocyte. Morphokinetic parameters analysis of embryos cultured in the Embryoscope imaging system was performed using the Embryoviewer software. According to the method of Ciray et al. [28], the kinetic parameters for each developmental stage of the blastocyst were manually marked. The embryonic development start time (t0) is the time of insemination. Each cleavage event of the embryo is timed at the moment of event completion. For example, t2 is the time at which the two blastomeres are completely separated. Morphokinetic parameters analyzed in this study included the appearance of two pronuclei (tpna), pn fade time (tpnf), division to two cells (t2), and subsequently to three cells (t3), four cells (t4), five cells (t5), and eight cells (t8), blastocyst initiation time (tsb) and blastocyst formation time (tb). Time interval parameters include the second cell cycle (cc2 = t3 – t2), the third cell cycle (cc3 = t5 – t3), and second synchrony (s2 = t4 – t3) as the time from division from a three-blastomere embryo to division into a four-blastomere embryo and third synchrony (s3 = t8 – t5) were also recorded.

### Sample amplification and classification of ploidy

The PGT cycles used in this study were all next-generation sequencing (NGS). Details of the NGS analysis procedure have been described previously [13]. Briefly, samples were amplified using a single-cell whole genome amplification (WGA)-based multiple annealing and cycle-based amplification cycling (MALBAC) protocol following a commercial kit protocol from Yikon Genomics. A series of DNA fragmentation, amplification, labeling, and purification were completed. Then, the product is purified. The final library was sequenced at approximately 0.04 × genome depth using the Life Technologies Ion Proton platform. This sequencing throughput produces reproducible copy number variations (CNVs) at ~ 4 MB resolution to detect variants. The threshold for aneuploidy detection was set to be greater than 70%. Thresholds for mosaic detection vary by chromosome. The lower limit of chromosomes 13, 16, 18, and 21 is 30%, the lower limit of chromosome 19 is 50%, and the other is 40%. Values below the lower limit indicate euploidy.

### Statistical analysis

All data analyses were performed using the Statistical Package for Social Sciences, version 13.0 (SPSS). Between-group data were analyzed using the nonparametric Mann–Whitney U test. Continuous data (t2, t3, …, tb) were compared using one-way ANOVA. The chi-square test was used to compare categorical data. Statistical significance was established at *P* < 0.05.

## Results

As shown in Table 1, a total of 405 PGT cycles were included in this study, and a TL monitoring culture was performed in each cycle. In 3 cycles, the patient gave up PGT. 360 cycles had blastocyst formation. The mean age of the patients was 31.2 years, the total number of retrieved oocytes was 5,736, and the mature oocytes were 4,551, with a 2pn rate of 73.1%. In total, 1467 blastocysts were available and biopsied. Among them, 537 (36.6%) euploid embryos, 691 (47.1%) aneuploid embryos, 225 (15.3%) mosaic embryos, and 14 blastocysts due to amplification failure have no data get.

**Table 1:** Clinical characteristics of PGT cycles

Parameter	
No. of cycles	405
Cancelled cycles (%)	3 (0.7)
Cycles with blastocysts	360
Age (y)	31.2 ± 4.5
Duration of infertility (y)	2.6 ± 2.4
Level of FSH	7.4 ± 2.5
Level of AMH	4.6 ± 3.7
BMI	22.1 ± 3.0
Time of ovarian stimulation (days)	10.0 ± 1.8
Total number of oocytes retrieved	5,736
Total number of matured oocytes	4,551
Total number of two pronucleus (2PN)	3,327
Fertilization rate (%)	73.1
Total available blastocyst formation	1,467
Average number of available blastocysts per cycle	3.65
No. of euploid (%)	537 (36.6)
No. of aneuploidy (%)	691 (47.1)
No. of mosaicism (%)	225 (15.3)
No. of amplification failed (%)	14 (0.9)

**Fig. 1:**
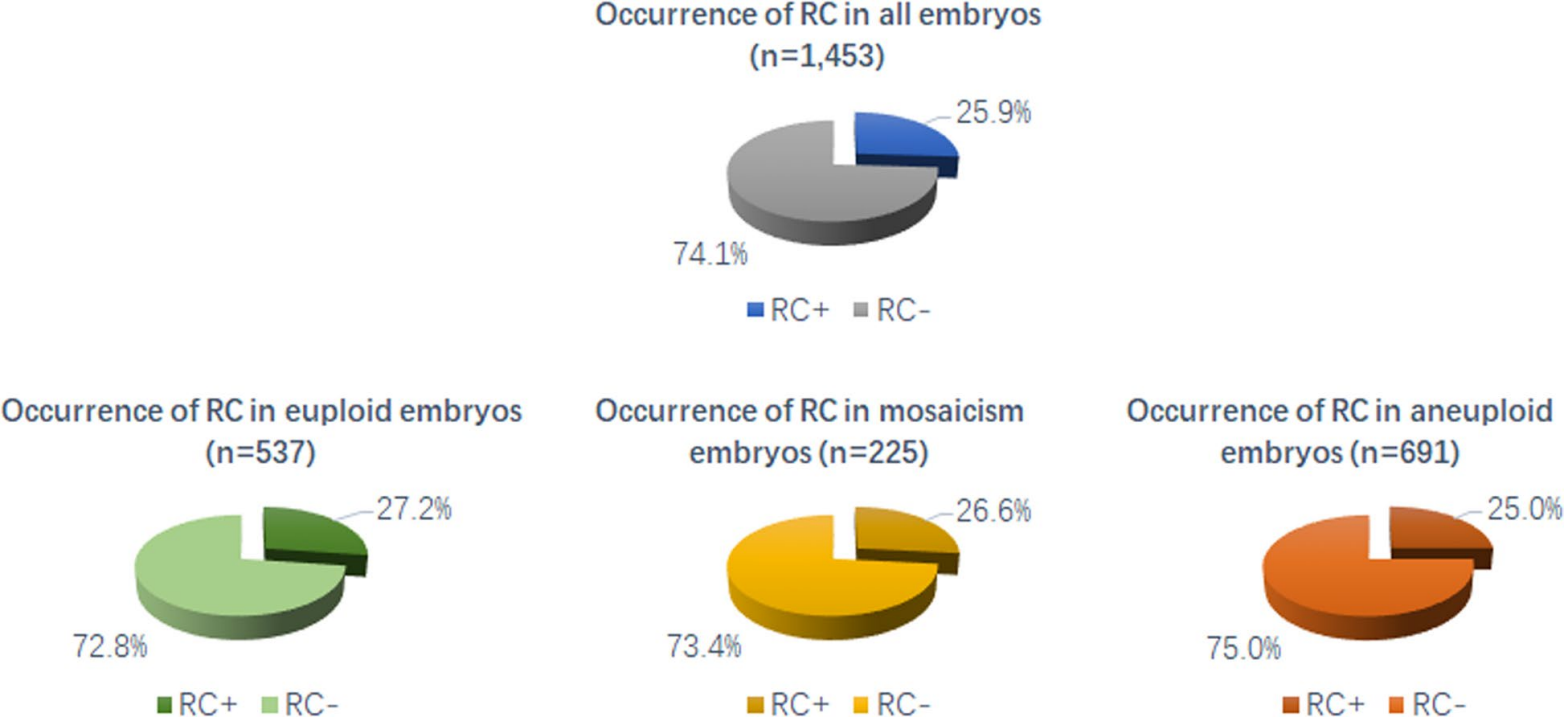
The occurrence of RC in all embryos and embryos of different ploidy groups. No statistically significant differences were found between the three groups. (euploid vs. aneuploid; euploid vs. mosaicism; aneuploid vs. mosaicism)

The results for the occurrence of RC in all embryos and embryos of different ploidy groups were presented in Fig. 1. Among the 1,453 blastocysts biopsied, 400 blastocysts showed RC phenomenon at the cleavage stage, and the incidence rate was 25.9%. In euploid, mosaic and aneuploid embryos, the incidence of RC was 27.2%, 26.6%, and 25.0%, respectively. The incidence of RC was similar among the three groups with no significant difference (*P* > 0.05).

**Fig. 2:**
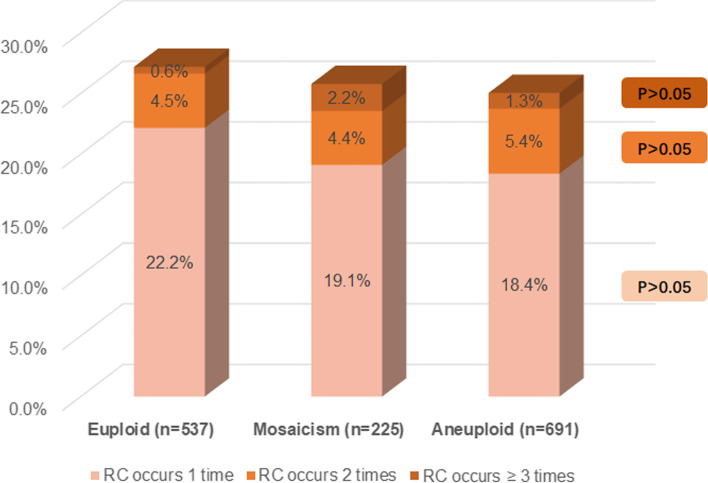
The relationship between the occurrence times and ploidy of RC during embryonic development. No statistically significant differences were found in the proportion of embryos with different occurrences of RC between the three groups. (euploid vs. aneuploid; euploid vs. mosaicism; aneuploid vs. mosaicism)

For the relationship between the occurrence times and ploidy of RC during embryonic development, the results were shown in Fig. 2. The proportions of RC occurrence one time, two times, and three or more times were generally similar among the euploidy, mosaic and aneuploidy groups (*P* > 0.05). Among these three groups, the proportion of embryos with one occurrence of RC was 18.4%-22.2%, the proportion of embryos with two occurrences of RC was 4.4%-5.4%, and the proportion of embryos with three or more occurrences was 0.6% %-1.3%. Overall, the number of RC occurrences was not associated with embryo ploidy status.

**Fig. 3:**
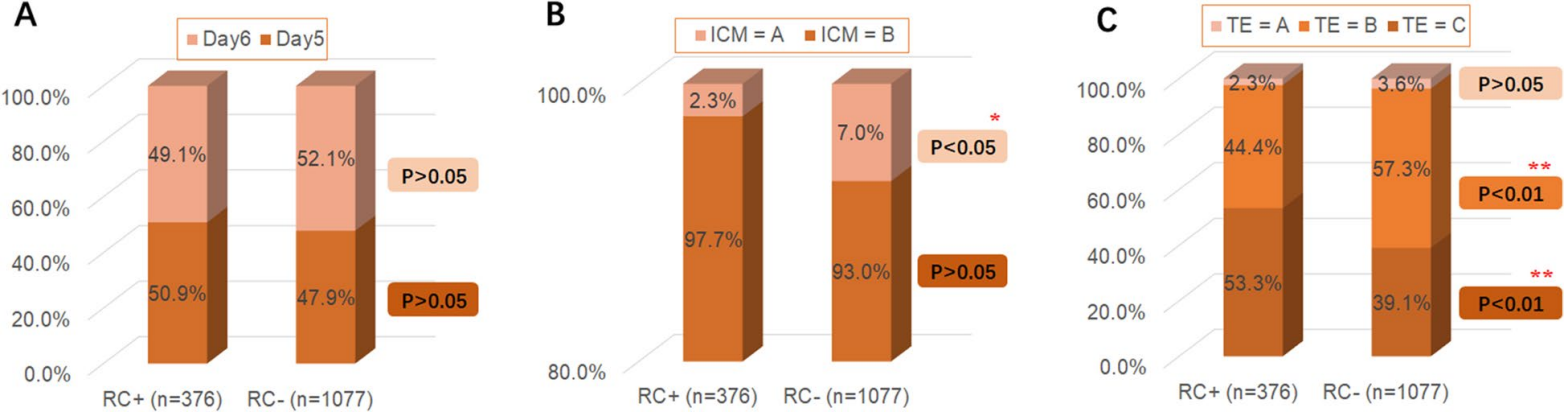
The relationship between the RC and the quality of blastocyst formation. There are significant differences in blastocyst quality between the RC + and RC- groups of embryos. (*, *P* < 0.05; **, *P* < 0.01)

The results of the relationship between the RC and the quality of blastocyst formation were shown in Fig. 3. According to the Gardner scoring system, among all embryos, there was no significant difference in the proportion of blastocysts formed on Day 5 and those formed on Day 6 between the group with RC (RC +) and the group without RC (RC-) (Fig. 3A). In terms of ICM score (Fig. 3B), the proportion of blastocysts graded as A in the RC + group was significantly lower than that in the RC- group (*P* < 0.05). In terms of TE score (Fig. 3C), the blastocysts rated A had no significant difference between the two groups, but blastocysts rated B were significantly lower in the RC + group than in the RC- group (*P* < 0.01). In general, the blastocyst quality of the RC + group was lower than that of the RC- group.

**Fig. 4:**
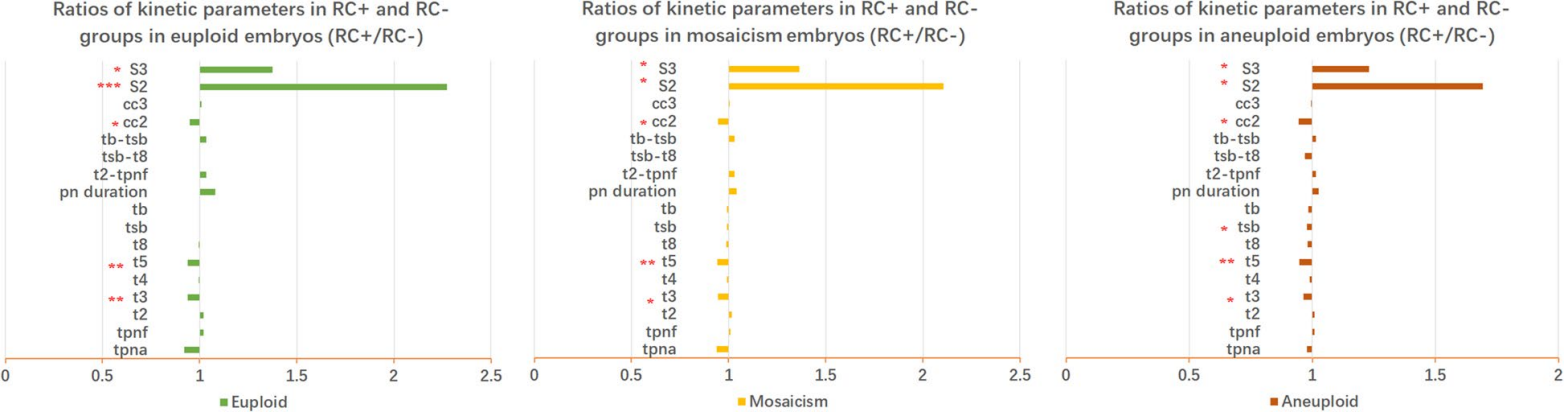
The results for the relationship between RC and kinetic parameters of embryonic development. There are significant differences in kinetic parameter values between the RC + and RC- groups of embryos. (*, *P* < 0.05; **, *P* < 0.01; ***, *P* < 0.001)

The results for the relationship between RC and kinetic parameters of embryonic development were shown in Fig. 4. For a more intuitive expression, the results were expressed as the ratio (RC + /RC-) of the kinetic parameters of the two groups of embryos. A ratio greater than 1 indicates that the kinetic parameters of the RC + group were longer than those of the RC- group. Time parameters t3, t5, and time interval parameter cc2 were significantly shorter in RC + embryos than in RC- embryos. The cleavage synchrony parameters s2 and s3 were significantly longer in RC + embryos than in RC- embryos. However, these changes in kinetic parameters were not significantly different between the euploid, mosaic and aneuploid groups.

## Discussion

This study aimed at RC, an abnormal phenomenon in embryonic development, and analyzed its relationship with the ploidy state of embryos. We hope to obtain an answer to whether the RC phenomenon affects the ploidy of embryos. This can give reproductive clinicians and embryologists a clear reference. We analyzed TL and PGT data of 1,467 blastocysts and found overall that the occurrence of cleavage-stage RC affected blastocyst developmental quality, but it did not affect blastocyst ploidy status (Fig. 1).

The RC phenomenon we often encounter in the process of clinical embryo selection. The overall incidence of RC in this article was 25.9%. It can be seen that the RC incidence rate is not low. This result is also similar to a previous report on RC. In the study by Liu et al. [19], the incidence of RC was 27.4%, and their results showed that RC significantly impairs embryonic developmental potential. This study by Liu et al. is the first report on the effect of RC on embryonic development. This result is essential, allowing us to recognize RC and its impact on embryonic development, and allowing embryologists to understand more about embryonic development. This is thanks to the application of TL technology, otherwise, we will never see this phenomenon. Liu’s results instruct embryologists to avoid selecting embryos with the RC phenomenon during embryo selection, which is essential for embryo laboratory work. However, for the ploidy study at the blastocyst stage, Liu’s results do not cover. To the best of our knowledge, there are few studies on the RC phenomenon and embryo ploidy status, and only one study has reported on this topic so far. Desai et al. analyzed the relationship between the occurrence of RC and the ploidy state of embryos, and the results showed that RC did not affect the ploidy state of embryos [29]. However, in the study by Desai et al., only 19 blastocysts were biopsied in total. We believe that this result needs more data validation, which is the purpose of our study. Because, if we find that the presence of RC leads to an increase in the rate of aneuploid or mosaicism, then embryologists need to be more careful when selecting embryos when encountering RC. On the contrary, this study confirmed that RC did not increase the aneuploidy rate or mosaicism rate of embryos through the results of 1467 blastocysts. In addition, we think that this result can be used as a reference not only for PGT cycles but also for routine IVF and ICSI patients. In conventional IVF and ICSI cycles, if RC appears at the cleavage stage, we can further screen embryos by extending the culture to blastocysts.

On the other hand, although the appearance of RC does not affect the ploidy state of blastocysts, it affects the latent blastocyst development potential. This is something we cannot ignore. The cleavage behavior in the cleavage stage is essential and directly affects the later development. RC directly affects the single-cell genome of human embryos, resulting in embryonic developmental arrest and reduced blastocyst formation rates [30–32]. There are several reports in the literature that abnormal cleavage patterns at the cleavage stage can impair the developmental potential of embryos, which in turn may affect the success rate of implantation. In a prospective observational study, 139 IVF cycles were recruited to assess the implantation efficiency of abnormally cleaved embryos. In embryos with confirmed implantation results, the implantation rate decreased from 67.0% to 0%. This result suggests that cleavage patterns can affect the developmental potential of day 3 human embryos [33]. Balakier et al. [20] reported only one live birth in 15 thawed embryo transfer (FET) cycles containing one RC embryo and one normal embryo. Barrie et al. [21] retrospectively analyzed 61 IVF cycles with RC occurrence, of which 9 cases had clear implantation results, including 3 cases of Day3 embryo transfer and 6 cases of Day5 embryo transfer, all of which were implantation failure, and the implantation rate was 0%. Liu et al. [19] transplanted 22 cases of RC embryos, the implantation rate was 4.5%, but no fetal heart was found in B-ultrasound. However, some studies reported that RC did not affect embryonic development. An examination of 1,698 embryos by Hickman et al. [24] found a prevalence of RC of 6.8%. Embryos appear to have similar fragmentation, cellularity, and morphokinetic characteristics compared to non-RC embryos. The authors concluded that RC does not appear to impair embryonic development to the blastocyst stage. This result was also supported by two other studies [25, 29]. Unfortunately, none of the three dissenting studies addressed the effect of RC embryos on implantation potential. Therefore, according to the data currently available, it is generally believed that RC will affect the implantation potential of embryos.

The results of this study showed that RC embryos showed a decrease in blastocyst quality. Unfortunately, we were unable to obtain data on the outcomes of these blastocysts. Because it is stipulated in the PGT cycle of our reproductive center that euploid embryos must be preferentially transferred, it is routinely preferred to transfer the one with the best quality score among the euploid embryos. As a result, very few blastocysts were transferred to RC in our samples. The acquisition of this data may require accumulation over a long period. This is a limitation of this study.

RC, as an abnormal division (Supplement Figure 1), refers to the refusion of two separate cells into one cell before 8 cells. Some researchers believe that the refusion between cells may be related to the abnormality of the cell membrane, which can lead to polyploidy and chromosomal mosaicism [20]. These abnormal fusions may also be affected by factors such as pH, temperature, and osmotic pressure difference [21]. Because in different embryo culture chambers, different medium components, and different buffer systems are used, the pH value and osmotic pressure after CO2 balance may be different. In addition, in the process of embryo culture, the level of oxygen concentration (5% or 20%), as well as the difference in temperature (37℃ or 36.5℃), the altitude of the laboratory, and the atmospheric pressure may give different results.

Furthermore, RC at the cleavage stage did not ultimately affect the ploidy outcome of blastocysts, and we speculate that this may be related to the repair mechanism during embryonic continuous mitosis. Embryos continuously screen themselves during this process. The developmental block of abnormal embryos eliminated most chromosomally abnormal embryos and screened out some euploid embryos with developmental potential. Relevant studies have also confirmed that embryos with developmental arrest have a higher rate of aneuploid [34]. The embryo also has the function of self-correction. Abnormal cleavage mainly results in an increase or decrease in the genetic material of daughter cells, and such abnormal daughter cells cannot be normally fused during cell compaction and are excluded. That is, euploidy of chromosomes is achieved by excluding chromosomally abnormal cells. Such cleavage protection mechanisms are not only found in human embryos but also in animal embryos [35].

In terms of embryonic cleavage, our parameters of embryonic development kinetics also gave us a phenomenon not observed before. Whether euploid, mosaic or aneuploid, embryos with RC exhibited poor cleavage synchrony (s2 and s3) (Fig. 4). It has been reported that the synchrony of cleavage is an important indicator of blastocyst formation and quality [8, 36–38]. Our results agree with this as well. The blastocyst quality in the RC + group was lower than that in the RC- group.

In conclusion, our study found that the chromosomal euploidy of cleavage-stage embryos with RC phenomenon developed to the blastocyst stage was not significantly different from that of cleavage normal blastocysts. Therefore, normally, in embryo transfer or cryopreservation, embryos with normal cleavage are preferentially selected; while for RC embryos, it is recommended to select and utilize blastocyst culture, which has similar clinical value to normal cleavage embryos. Although the mechanism and impact of tripolar cytokinesis or cell fusion occurring at reverse cleavage remains uncertain, this at least suggests that embryonic plasticity is strong. As Coticchio et al. stated, in future long-term studies, the correlation between abnormal cleavage embryos and neonatal information can tell us more. In the future, more basic research on abnormal cleavage and AI-related big data analysis may also challenge current concepts and may rewrite new rules governing cell cycle, cell determination, and chromosome segregation. The human embryo is placed in a preeminent position in the field of developmental biology.

## Supplementary Information

**Additional file 1.** Reverse Cleavage

**Additional file 2:** **Supplement Table 1.** Timelapse assessment results for euploidy, mosaicism and aneuploidy group.

## Data Availability

The datasets used and/or analyzed during the current study are available from the corresponding author on reasonable request.
