# Supplementary figures and images for: Incidence, dynamics and recurrences of reverse cleavage in aneuploid, mosaic and euploid blastocysts, and its relationship with embryo quality
Source: J Ovarian Res. 2022 Aug 5;15:91. doi: 10.1186/s13048-022-01026-9 (PMC9356443; doi:10.1186/s13048-022-01026-9)

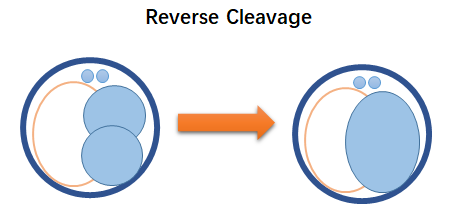

Supplement: Supplementary file 1 — Additional file 1. Reverse Cleavage [file 13048_2022_1026_MOESM1_ESM.tif]
