# Supplementary material for: Incidence, dynamics and recurrences of reverse cleavage in aneuploid, mosaic and euploid blastocysts, and its relationship with embryo quality
Source: J Ovarian Res. 2022 Aug 5;15:91. doi: 10.1186/s13048-022-01026-9 (PMC9356443; doi:10.1186/s13048-022-01026-9)
Supplement: Supplementary file 2 — Additional file 2: Supplement Table 1. Timelapse assessment results for euploidy, mosaicism and aneuploidy group. [file 13048_2022_1026_MOESM2_ESM.docx]

**Supplement Table 1.** Timelapse assessment results for euploidy, mosaicism and aneuploidy group.

| Kinetic parameter | Euploid (hours) | | Sig. |  | Mosaicism (hours) | | Sig. |  | Aneuploid (hours) | | Sig. |
| --- | --- | --- | --- | --- | --- | --- | --- | --- | --- | --- | --- |
|  | RC+ | RC- |  |  | RC+ | RC- |  |  | RC+ | RC- |  |
| tpna | 8.0±2.0 | 8.7±2.1 |  |  | 8.1±2 | 8.6±2 |  |  | 8.0±2.0 | 8.1±1.9 |  |
| tpnf | 23.2±3.0 | 22.7±2.7 |  |  | 23±2.8 | 22.8±2.7 |  |  | 22.8±2.7 | 22.6±2.6 |  |
| t2 | 25.6±3.2 | 25.1±2.7 |  |  | 25.3±2.8 | 25±2.7 |  |  | 25.4±2.8 | 25.1±2.7 |  |
| t3 | 33.6±5.4 | 35.8±3.9 | ** |  | 33.9±5.2 | 35.8±4.7 | * |  | 34.6±4.6 | 35.9±4 | * |
| t4 | 36.6±3.9 | 36.7±3.5 |  |  | 36.6±4.4 | 36.9±3.9 |  |  | 36.6±4.5 | 37.0±4.0 |  |
| t5 | 44.9±7.7 | 47.9±6.2 | ** |  | 45.9±7.5 | 48.7±6.2 | ** |  | 46.5±7.6 | 49±6.1 | ** |
| t8 | 54.8±8.1 | 54.9±7.2 |  |  | 54.1±7.7 | 55±7.8 |  |  | 55.0±7.9 | 56.1±8.5 |  |
| tsb | 97.4±7.8 | 97.3±8.7 |  |  | 98±7.9 | 98.6±8.6 |  |  | 97.5±7.9 | 99.5±8.4 | * |
| tb | 108.1±8.6 | 107.7±9.2 |  |  | 108.2±8.4 | 108.4±9.4 |  |  | 108.8±9.0 | 110.6±9.1 |  |
| pn druation | 15.2±2.9 | 14±2.8 |  |  | 15±2.9 | 14.4±2.5 |  |  | 14.9±2.7 | 14.5±2.6 |  |
| t2-tpnf | 2.4±0.6 | 2.4±0.4 |  |  | 2.5±0.4 | 2.5±0.4 |  |  | 2.5±0.3 | 2.5±0.4 |  |
| tsb-t8 | 42.7±7.8 | 42.5±8.0 |  |  | 42.8±7.7 | 42.8±7.8 |  |  | 41.8±8.1 | 43.0±8.2 |  |
| tb-tsb | 10.7±3.5 | 10.4±3.8 |  |  | 10.7±4.1 | 10.7±4.4 |  |  | 11.3±4.1 | 11.1±4.5 |  |
| cc2 | 11.1±1.7 | 11.7±1.8 | * |  | 11.1±2.2 | 11.8±2.1 | * |  | 11.3±2.9 | 11.9±2.1 | * |
| cc3 | 18.4±7.0 | 18.2±5.7 |  |  | 18.8±7.2 | 18.7±7 |  |  | 19.1±6.5 | 19.2±6.6 |  |
| s2 | 2.7±3.2 | 1.2±2.2 | *** |  | 2.6±3.4 | 1.3±2.4 | * |  | 2.1±3.1 | 1.3±2.0 | * |
| s3 | 9.9±8.0 | 7.2±6.7 | * |  | 9.7±7.5 | 7.1±6.4 | * |  | 9.0±6.9 | 7.3±6.7 | * |

There are significant differences in kinetic parameter values between the RC+ and RC- groups of embryos. (*, P<0.05; **, P<0.01; ***, P<0.001 )
